# The International Society of Gynecological Pathologists (ISGyP) Endometrial Carcinoma Project

**DOI:** 10.1097/PGP.0000000000000495

**Published:** 2018-12-14

**Authors:** W. Glenn McCluggage, Anais Malpica, Xavier Matias-Guiu, Esther Oliva, Vinita Parkash

**Affiliations:** Department of Pathology, Belfast Health and Social Care Trust, Belfast, UK (W.G.M.); Department of Pathology, The University of Texas MD Anderson Cancer Center, Houston, Texas (A.M.); Departments of Pathology, Hospital University Arnau de Vilanova and, Hospital University de Bellvitge, IRBLLEIDA, Idibell, University of Lleida, CIBERONC, Barcelona, Spain (X.M.-G.); James Homer Wright Pathology Laboratories, Massachusetts General Hospital, and Harvard Medical School, Boston, Massachusetts (E.O.); Department of Pathology and Obstetrics and Gynecology, Yale School of Medicine and Yale School of Public Health, New Haven, Connecticut (V.P.)

Endometrial carcinoma is the commonest gynecological malignancy in developed countries, and the various aspects of the pathology report are critical for patient management. There are many areas of controversy with regard to the handling of resection specimens and the pathologic reporting of endometrial carcinomas. These controversies include those related to sampling, diagnosis, reporting of parameters important for staging, and the undertaking of ancillary studies. These controversies stimulated the International Society of Gynecological Pathologists (ISGyP) endometrial carcinoma project. The project was devised at the ISGyP Board of Directors meeting in March 2015 under the Presidency of Richard Zaino. An organizing committee was selected from the members of the Board of Directors and the education committee of the ISGyP. The organizing committee (comprising the 5 authors of this editorial), as a first step, devised a comprehensive survey, which was emailed to all members of the ISGyP; the survey covered all aspects of endometrial cancer reporting, including specimen dissection and sampling, diagnosis, staging, prognostic factors, and ancillary studies.

Following the survey, the members of the organizing committee devised a series of key questions chosen to encompass the various areas of controversy. These questions were divided into three major groups (a-Diagnosis; b-Processing, Sampling, Staging, and Prognosis; c-Special Techniques/Ancillary Studies). For each group, 5 “lead” pathologists were selected by the organizing committee, and ∼15 other pathologists were selected for each group to work on the various questions. Usually, 3 pathologists worked on each question, their task being to critically review the literature and to come up with recommendations, which were to be “evidence-based” if possible, although it was recognized that this was not feasible when the levels of evidence were weak. A commentary with selected important references was produced for each question. Many of the recommendations were presented and discussed at an ISGyP-sponsored symposium in Seattle before the United States and Canadian Academy of Pathology meeting in March 2016; some of the recommendations and commentaries were amended following these discussions.

The recommendations and associated commentaries are presented in the various articles in this issue of *International Journal of Gynecological Pathology*. The first article details the results of the survey undertaken before the start of the project. This provides important background information with regard to current practices worldwide. The second article covers the important issue of sampling of resection specimens of endometrial carcinomas. The following 3 articles deal with various aspects related to the diagnosis of the different morphologic subtypes of endometrial carcinoma, the sometimes problematic distinction between an endometrial and a cervical carcinoma, and the issue of synchronous endometrial and adnexal carcinomas. An important recommendation is that endometrioid carcinomas be graded using a binary International Federation of Gynecology and Obstetrics grading system rather than the current 3-tier International Federation of Gynecology and Obstetrics grading system. Advice is provided as to how to incorporate the 4 genomic subcategories of endometrial carcinoma, as identified through The Cancer Genome Atlas Study, into clinical practice. The next article deals with parameters that are important in the staging and prognosis of endometrial carcinomas and provides recommendations with regard to the many controversial issues.

The final 2 articles cover ancillary studies with a recommendation that all endometrial carcinomas be tested for mismatch repair protein abnormalities and possible Lynch syndrome using immunohistochemistry for mismatch repair proteins as the initial modality. Given the wide usage of p53 immunohistochemistry in the diagnosis of endometrial carcinomas and the well-known problems in the interpretation of p53 staining, a separate article is devoted to p53 immunohistochemistry; this provides important recommendations with regard to the methods of p53 immunohistochemical staining and interpretation.

It has been a great pleasure for us to organize this project and to help in coordinating and writing the various articles. We are thankful to all the pathologists who participated in the project and the authors of the various articles. We hope the articles will serve as important reference material for pathologists currently and in the future and aid in the standardization of sampling, diagnosis, and reporting of endometrial carcinomas, which will benefit patients with this disease. We recognize that this is a rapidly evolving field, and regular updating of these recommendations will be necessary in the future.

